# Research on equity analysis and forecasting of nursing human resource allocation in Jiangxi Province, China

**DOI:** 10.1016/j.ijnss.2024.12.009

**Published:** 2024-12-19

**Authors:** Yunyu Du, Zhiqin Xie, Zhen Yang, Wanyin Xiong, Li Zhou, Min Zhang, Suhua Zeng, Min Wang

**Affiliations:** a Department of Thoracic Surgery, The First Affiliated Hospital, Jiangxi Medical College, Nanchang University, Nanchang, Jiangxi, China; b Department of Nursing, The First Affiliated Hospital, Jiangxi Medical College, Nanchang University, Nanchang, Jiangxi, China; c Jiangxi Medical Center for Critical Public Health Events, The First Affiliated Hospital, Jiangxi Medical College, Nanchang University, Nanchang, Jiangxi, China

**Keywords:** Forecasting, Equity, Nursing administration, Nursing human resource

## Abstract

**Objectives:**

This study aimed to assess the equity of nursing human resource allocation in Jiangxi Province, China, and forecast future trends in the next five years.

**Methods:**

We used the related data from the *China Statistical Yearbook*, *China Health Statistics Yearbook*, and *Jiangxi Statistical Yearbook* (2003–2022). The equity of nursing human resource allocation was evaluated using Lorenz curves, Gini coefficients, and Theil index, from the perspective of population and geographical area. Demands for nursing human resource in Jiangxi Province from 2023 to 2027 were forecasted using the Autoregressive Integrated Moving Average (ARIMA) and Grey (1,1) models.

**Results:**

From 2003 to 2022, all the key nursing human resource indicators continuously increased; the number of registered nurses in Jiangxi Province increased by 109,786, with an average annual growth rate of 7.80%. Registered nurses per 1,000 population rose by 2.21, while nurses per square kilometer increased by 0.66. Jiangxi Province has surpassed the national level in several nursing resource indicators, including registered nurses as a percentage of health technicians, registered nurses per square kilometer, and doctor-to-nurse ratio. Within the province, all indicators in cities are higher than those in county-level regions. Among the cities in Jiangxi Province, Ganzhou City had the highest number of registered nurses, Xinyu City led in the doctor-to-nurse ratio, and Nanchang City had the highest bed-to-nurse ratio. In 2022, the Gini coefficients for registered nurses in Jiangxi Province were 0.09 by population and 0.34 by geographical area, reflecting the allocation of registered nurses in Jiangxi Province is highly equitable by population but relatively equitable by geographical area. Forecasting results suggested that the number of registered nurses in Jiangxi Province will reach 170,100 by 2027, indicating continued growth and improvement in nursing resource allocation.

**Conclusions:**

Over the past two decades, the human nursing resources in Jiangxi Province have grown substantially. The absolute fairness of nurse human resources allocation by population highlights significant progress, although regional disparities persist. These findings provide a foundation for optimizing future nursing resource allocation to ensure equitable access to healthcare services.

## What is known?

• Jiangxi Province has entered the stage of mild aging, with its population accounting for 3.2% of China’s total population. However, disparities in population size and economic development exist in its area, leading to varying demands for health services.
• Equitable distribution of health resources is fundamental for achieving population health equity, ensuring that all regions can effectively meet their specific healthcare needs.

## What is new?

• Over the past two decades, nursing resources in Jiangxi Province have grown substantially.
• The absolute fairness of nurse distribution by population highlights significant progress, although regional disparities persist.

## 1 Introduction

Nursing human resources comprise the personnel engaged in nursing work across all levels of medical institutions [1]. Adequate and equitable allocation of these resources is crucial for ensuring patient safety, improving the quality of nursing care, and enhancing the overall healthcare experience. As China and other nations face an aging population, one key challenge in elderly care is the need for qualified nursing personnel [2]. However, both globally and within China, nursing resources still need to be more distributed among regions [3]. In line with the Global Strategy on Human Resources for Health: Workforce 2030, WHO estimates a global shortfall of over 18 million healthcare workers [4]. The persistent shortages expected in the future vary widely across countries [3,5]. The causes of these shortages are multifaceted. Poor working conditions, including workplace violence and low social status, contribute to job dissatisfaction. Imbalances in workforce composition—such as a predominantly young and female workforce—further exacerbate the issue. Low-income levels, limited development opportunities, narrow scopes of practice, and insufficient policy support compound the challenges nurses face. High stress levels and job burnout are additional factors undermining the stability of the nursing workforce [1,3]. WHO emphasizes fairness, efficiency, and utility as ethical principles guiding health resource allocation [6]. The nursing workforce’s size, distribution, and composition are key determinants of healthcare quality and efficiency [3]. Recognizing this, the National Health Commission of China has called for improved management of nursing resources and establishing a high-quality, efficient nursing service system [4]. At the hospital level, nurse staffing significantly influences institutional performance and patient outcomes [7]. This became particularly evident during the COVID-19 pandemic, highlighting the critical role of national health resources as a safety net [8]. Addressing the global nursing workforce gap and ensuring equitable resource allocation is vital to promoting global health equity and resilience.

Recently, equitable distribution of nursing resources has gained attention in research. A study suggests a link between health resource equity and economic development, with high-income regions focusing on efficient utilization while low-income areas struggle with resource accessibility [9]. According to the National Bureau of Statistics, Jiangxi Province’s 2022 gross domestic product (GDP) places it in the fourth economic tier, reflecting its relative underdevelopment compared to other regions in China [10]. Furthermore, the population of Jiangxi Province accounts for 3.2% of China’s total population. The population and economic development could be more balanced among the prefecture-level cities, and the demand for health services is also different. At the same time, the population of 60 years old and above in Jiangxi Province is 7,624,800, accounting for 16.87% of the total population, which has entered the stage of mild aging [11]. The rational allocation of nursing human resources is of great significance to solving the health service needs of residents in Jiangxi Province and promoting realizing the goal of nursing human resource allocation. This underscores the need for better strategies to optimize health resource allocation. Unfortunately, the last study was published in 2009 [12], the data was old and incomprehensive, so it is difficult to judge the rationality of the current medical human resource allocation while providing a reference for the scientific formulation of medical and health development plans.

To address these gaps, this study aimed to evaluate the fairness of nursing resource distribution in Jiangxi Province by examining some key indicators and forecasting them to provide a reference for the scientific formulation of medical and health development plans and promote the rational allocation of nursing human resources in Jiangxi Province.

## 2 Methods

### 2.1 Data sources

This study utilized publicly available data from the *China Statistical Yearbook*, *China Health Statistics Yearbook*, and the *Jiangxi Statistical Yearbook* (2003–2022) [10,13]. These datasets include information on the permanent resident population, gender distribution, urban and county populations, and healthcare statistics such as the number of registered nurses, practicing (assistant) doctors, health technicians, and hospital beds in Jiangxi Province. In this study, we focused on some key indicators, including the total number of registered nurses, number of registered nurses per 1,000 population, number of registered nurses per square kilometer, doctor-to-nurse ratio, bed-to-nurse ratio, the proportion of nurses within the health technicians, and the average annual growth rate of nurses. The growth rate was calculated using the formula: $\left(\sqrt[{n}]{\frac{a_{n}}{a_{0}}}\;-\;1\right)$ × 100%, *n* is the year of growth, *a*_*n*_ is the number of registered nurses in the growth year, *a*_*0*_ is the number of registered nurses in the initial year. As these sources provide aggregated and anonymized data, direct ethical approval was not required according to institutional guidelines.

### 2.2 Equity analysis

#### 2.2.1 The Lorentz curve

The Lorentz curve was proposed by the American statistician (Lorentz) in 1905 to compare and analyze the equality of income and wealth in a country at different times or in other countries in the same era. It has also been widely applied to evaluate the equity of health resource allocation. The Lorentz curve can intuitively and clearly reflect the equity of allocation. In the original figure of the Lorentz curve (Appendix A), there is an absolute fair line (45° diagonal). When the distance between the Lorentz curve and the absolute fair line is closer, representing the more equitable allocation of resources, and the curvature of the Lorentz curve is greater, the allocation of resources is more unfair [14]. In this study, we drew the Lorentz curve, with the X-axis representing the cumulative population and areas and the Y-axis representing the cumulative number of registered nurses.

**Fig. 1:**
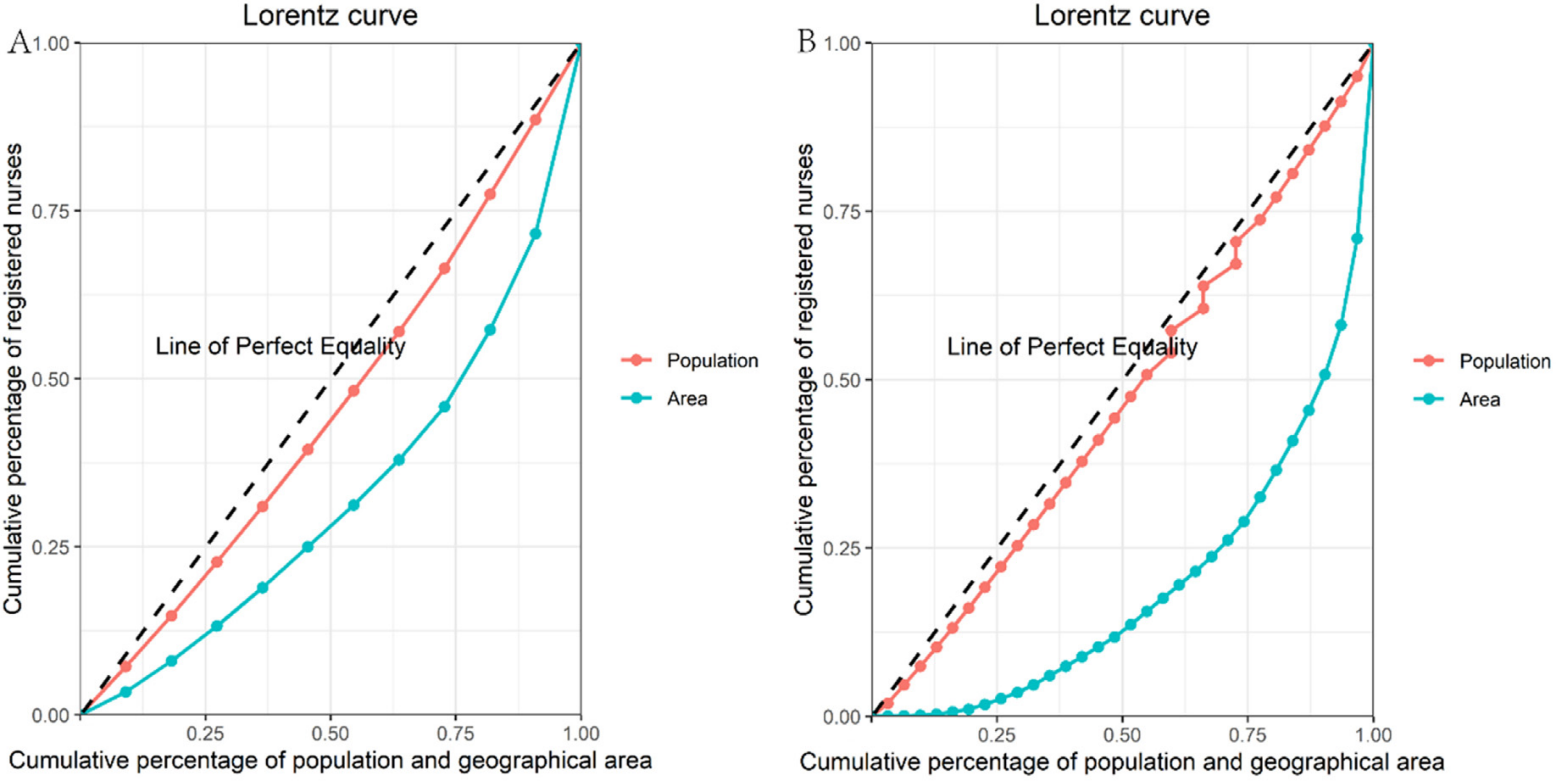
The Lorentz curve of registered nurses in Jiangxi Province (A) and China (B) in 2022.

#### 2.2.2 Gini coefficient

The Gini coefficient is a standard indicator calculated according to the Lorentz curve to measure whether the income level of people in a country or area is fair. The closer the Gini coefficient is to 0, the more equitable the allocation of resources. As shown in Appendix A, A represents the area between the absolute fair line and the Lorentz curve, B represents the area between the Lorentz curve and the X-axis, and the Gini coefficient is equal to A/(A + B). The Gini coefficient was categorized as follows: <0.2 (absolute fairness); 0.2–0.3 (moderate fairness); 0.3–0.4 (relative fairness); >0.4 (warning of inequality); >0.6 (high inequality) [15,16]. This method systematically quantifies inequality by comparing cumulative resource distributions across regions relative to population proportions.

The Gini coefficient is calculated as G = 1-$\sum_{i=1}^{n}W_{i}\left(2V_{i}-Y_{i}\right)$. The Gini coefficient (G) measures inequality, calculated using the following variables: *n* is the total number of regions (e.g., cities in Jiangxi Province). *i* is the ranking of regions based on per region resource ownership, arranged from lowest to highest (*i* = 1, 2, 3 … *n*). *W*_*i*_ is the proportion of the population in a city relative to the total population of Jiangxi Province. *V_i_* is the cumulative percentage of registered nurses per 1,000 population. *Y_i_* is the proportion of registered nurses in a city relative to the total number of registered nurses in Jiangxi Province.

#### 2.2.3 Theil index

The Theil index was proposed by Dutch economist (H. Theil) [17] to investigate the difference from the perspective of information quantity and entropy. The Theil index represents the overall difference and can reflect the differences between regions and within regions. In addition, it can clearly show each and the overall region and has a certain complementarity and the Gini coefficient [17,18]. The Theil index ranges from 0 to 1, the smaller the value, the smaller the difference in human resource allocation, and vice versa. The formula is as follows:

$$T=\frac{1}{n}\sum_{i=1}^{n}\frac{Y_{i}}{y}\ln\left(\frac{Y_{i}}{¯y}\right)$$

In the above formula, *n* is the total number of cities, *Yi* is the number of registered nurses in the first city, and y is the average number of registered nurses in 11 cities in Jiangxi Province. The overall differences reflected by the Theil index can be further decomposed into within-group difference *T*_*w*_ and between-group difference *T*_*B*_. The formula is as follows:

$$T=T_{w}+T_{B}$$

$$T_{w}=\sum_{p=1}^{m}\frac{Y_{i}}{y}\ln\left(\frac{n_{p}}{n}\frac{{¯e}_{p}}{¯e}\right)T_{p}:\;T_{B}=\sum_{p=1}^{m}\left(\frac{n_{p}}{n}\frac{{¯e}_{p}}{¯e}\right)\ln\left(\frac{{¯e}_{p}}{¯e}\right)$$

Calculate the contribution rate between cities and within cities to the overall differences: *T*_*w*_ Contribution rate = *T*_*w*_/*T, T*_*B*_ Contribution rate = *T*_*B*_/*T.*

### 2.3 Demand forecasting

#### 2.3.1 Autoregressive Integrated Moving Average model

The Autoregressive Integrated Moving Average (ARIMA) model has been widely used in forecasting health resource trends due to its easy operation and good prediction effect. The ARIMA model combines AR (autoregressive), I (integrated), and MA (moving average) to analyze time-series data. The formula of the ARIMA model is Y_*t*_ = _ϕ1_Y_*t−1*_ + _ϕ2_Y_*t−2*_ + … + _ϕp_Y_*t−p*_ + e_*t*_ − θ_*1*_e_*t−1*_ − … − θ_*q*_e_*t−q*_, where (_ϕ1_Y_*t−1*_ + _ϕ2_Y_*t−2*_ + … + _ϕp_Y_*t−p*_ + e_*t*_) is the AR model part, e_*t*_ − θ_*1*_e_*t−1*_ − … − θ_*q*_e_*t−q*_ is the MA model part, Y_*t−p*_ is the observed value at the period of (*t* − *p*) is the MA model part, *p* and *q* represent the model order of AR and MA, and e_*t*_ is the random error at the period of *t* [19].

The construction process of the ARIMA Model is as follows: 1) establish time series data: the time series was constructed using nursing human resource data from Jiangxi Province from 2003 to 2022; 2) assess stationarity: the time series was plotted to assess stationarity. If non-stationary, differencing was applied until a stationary series was achieved (determined by *d*); 3) determine optimal orders (*p, d, q*). The optimal autoregressive (*p*) and moving average (*q*) orders were identified using the auto. arima function in R’s ‘forecast’ package. The diffs function in R was used to determine the best difference order; 4) model validation: the final ARIMA (*p, d, q*) model was validated through comprehensive testing to ensure it accurately captured the characteristics of the data. This step confirms the model’s predictive reliability for forecasting trends of nursing resources in Jiangxi Province [20].

#### 2.3.2 Grey (1,1) model

The Grey prediction model, introduced by renowned scholar Julong Deng in the 1980s, was designed to address the uncertainty of small sample datasets. This model is particularly advantageous due to its minimal data requirements, high degree of fit, strong predictive performance, and practicality. The Grey model has been widely applied in healthcare to forecast various metrics, including the number of health personnel, medical expenses, disease incidence, and mortality rates. Its high predictive accuracy and robust performance make it particularly suitable for medium and long-term forecasting, supporting strategic planning in healthcare systems [21]. The characteristic of this model is not the need to know the distribution type of the data, and it can be fitted as long as the data is non-negative monotonic. It is based on the random original time series through the new time series formed after accumulation. It approaches using the solution of the first-order linear differential equation according to the law of the new time series.

$${\hat{X}}^{\left(1\right)}\left(k\right)=\left(X^{\left(0\right)}\left(1\right)-\frac{u}{a}\right)e^{-a\left(k-1\right)}+\frac{u}{a}$$

In equation *a* and *u* are the undetermined coefficients, where *a* is the developmental grey, and *u* is the endogenous control grey. We obtained the overdetermined equations about *a* and *u* by discretizing the differential equations and finding the parameter vector by the least squares method.

The accuracy of the model prediction was tested by the posterior difference ratio (*C*) and the slight error probability (*P*). If *C* < 0.35 and *P* ≥ 0.95, the accuracy of the model is level 1 (excellent); if 0.35 ≤ *C* < 0.50 and 0.80 ≤ *P* < 0.95 is level 2 (qualified); if 0.50 ≤ *C* < 0.65 and 0.70 ≤ *P* < 0.80 is level 3 (barely qualified); if *C* ≥ 0.65 and *P* < 0.70 is level 4 (unqualified). If the model accuracy reaches level 1 or level 2, the model can be used for extrapolation prediction. If the fitting accuracy of the two is not qualified, it cannot be directly used for extrapolation prediction and then extrapolation prediction after residual correction [22].

### 2.4 Statistical analysis

Microsoft Excel 2019 was used to compile and process the data. The Lorenz curve and Gini coefficient were calculated using R version 4.2.3, and the Theil index was calculated using Microsoft Excel 2019. The Grey model was performed using the Matlab 7.1 Grey model program, and the ARIMA model was constructed using the ‘forecast’ package in R alongside SPSS 23.0.

## 3 Results

### 3.1 Current situation of nursing human resource allocation

Since 2003, the key nursing human resource indicators in Jiangxi Province have increased continuously, as summarized in Appendix B. From 2003 to 2022, the total number of registered nurses increased by 109,786, with an average annual growth rate of 7.8%. The number of registered nurses per 1,000 population increased by 2.21, and per square kilometer rose by 0.66. The proportion of nurses within the healthcare staff increased by 0.17. The doctor-to-nurse ratio improved from 1:0.70 to 1:1.27, and the bed-to-nurse ratio increased from 1:0.41 to 1:0.46.

Jiangxi Province exceeds the national average level in the proportion of registered nurses as a percentage of health technicians, number of registered nurses per square kilometer, and doctor-to-nurse ratio. The bed-to-nurse ratio in Jiangxi Province exceeded the national average before 2013 and was equal in 2013 but was lower after 2014. However, other indicators are below the national level.

### 3.2 Current situation of nursing human resource allocation in various regions

The highest number of registered nurses was in Nanchang City (26,773), and the lowest was in Yingtan City (3,125). The city with the highest number of nurses per 1,000 population was Nanchang City (4.09), and the lowest city was Ji’an City (2.58). The city with the highest number of registered nurses per square kilometer was Nanchang City (3.72), and the lowest was Ji’an City (0.45). The most significant proportion of registered nurses among health technicians was in Xinyu City (0.50), while the lowest was in Ji’an City (0.42). The city with the highest doctor-to-nurse ratio was Ji’an City (1:1.09), and the lowest was Xinyu City (1:1.44). The lowest bed-to-nurse ratio is Ji’an City (1:0.36), while Nanchang City (1:0.59) is the highest. The details are summarized in Appendix B.

### 3.3 Comparison of urban and county regions’ registered nurse

The number of registered nurses in urban areas fluctuated over time but showed an upward trend, reaching a maximum of 74,665 in 2022. The percentage of registered nurses among health technicians in urban areas also exhibited a general increase between 2020 and 2022. Additionally, the number of registered nurses per 1,000 population in urban areas reached the highest point in 2017 at 5.27. In contrast, the above three indicators consistently increased in counties from 2003 to 2022. The details are summarized in Appendix B.

### 3.4 Equity analysis of nursing human resources allocation

As shown in Fig. 1A, the red curve representing the distribution of registered nurses based on population in Jiangxi Province closely aligns with the line of perfect equality. In contrast, the curve for area-based distribution deviates significantly. This suggests that the allocation of registered nurses in Jiangxi Province is highly equitable by population but only moderately fair by area. The Gini coefficient for the distribution of registered nurses in Jiangxi Province in 2022 was 0.09 when assessed by population and 0.34 by geographical area. In 2022, China’s Gini coefficient for registered nurses was 0.06 based on population and 0.61 based on regional area (Fig. 1B), indicating a high level of fairness in population distribution but significant inequality in regional distribution. The equity of registered nurse distribution in Jiangxi Province by population is lower than the national average. However, the province achieves a higher level of equity in regional distribution compared to the country as a whole.

From 2003 to 2022, the total Theil index of nursing human resources in Jiangxi Province was between 0.14 and 0.18. This indicates that the fairness of nursing human resources allocation in Jiangxi Province needs to be improved. From the perspective of the contribution rate of the Theil index, the contribution rate within the group was more significant than the contribution rate between the groups, indicating that the difference in nursing human resource allocation in Jiangxi Province was mainly caused by the group (Appendix B).

### 3.5 Forecasting analysis of nursing human resources

#### 3.5.1 ARIMA model

**Fig. 2:**
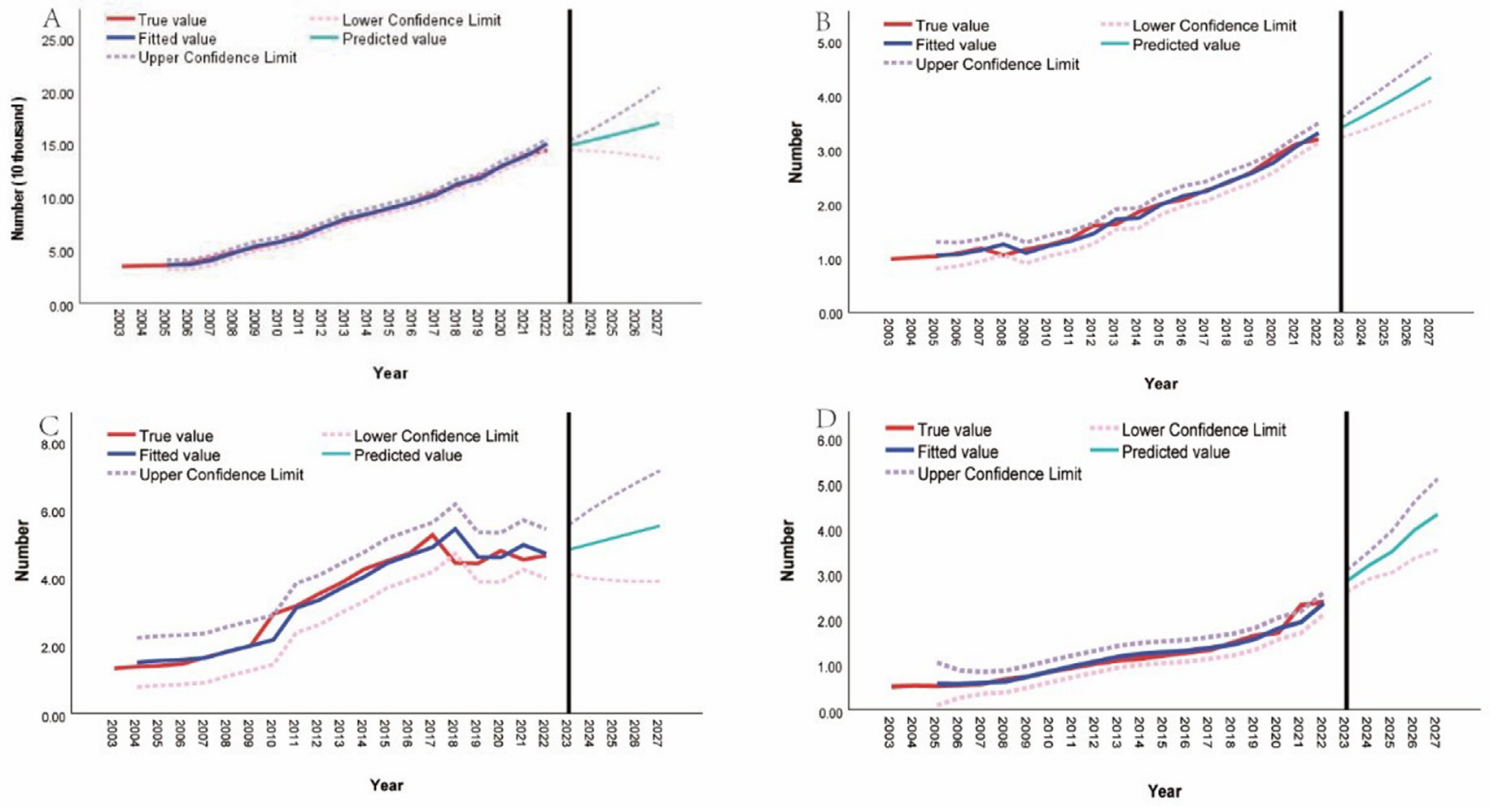
Actual value and predicted value of nursing human resources in Jiangxi Province from 2003 to 2027. A: total number of registered nurses; B: number of registered nurses per 1,000 population; C: number of registered nurses per 1,000 population in urban; D: registered nurses per 1,000 population in county.

A visual inspection of the time-series data (Appendix A) revealed a clear upward trend, indicating non-stationarity. To address this, differencing is required to stabilize the data for further analysis. After differencing, the data fluctuated around zero, suggesting stationarity (Appendix A). The differencing orders (*d*) for the four datasets—registered nurses, registered nurses per 1,000 population, registered nurses per 1,000 in county seats, and registered nurses per 1,000 in rural areas—were determined to be 2, 2, 1, and 2, respectively. Using the auto. arima function in R, the optimal ARIMA model parameters (*p, d, q*) were identified for each time series. The ARIMA model’s goodness-of-fit was substantial (Fig. 2). The projections suggest a continued upward trend in registered nurses per thousand population in Jiangxi Province, urban areas, and counties through 2027. By 2027, the total number of registered nurses is expected to reach 170,100, with nurses per 1,000 population rising to 3.76, reaching 5.54 in urban areas and 4.32 in counties (Appendix B).

#### 3.5.2 Grey (1,1) model

Through the Matlab7.1 Grey model program, the values of parameters a and u can be obtained and returned to formula 1 to get the number of registered nurses in Jiangxi Province, the number of registered nurses per 1,000 population, the number of registered nurses per 1,000 population in urban areas, and the number of registered nurses per 1,000 population in the county seat. The test results of the model are shown in Appendix B. At the same time, according to the test statistics *C* and *P*, the four index model fits are determined to be unqualified, and the grade is level 4. While the grey model projects that the total number of nurses in Jiangxi Province will reach 187,500 by 2027, the model’s extrapolation accuracy needs to meet the required standards. Therefore, the reliability of these predictions is limited. The detailed results are presented in Appendix B.

## 4 Discussion

The equitable and efficient allocation of health resources is fundamental to improving public welfare and advancing the health status of a country or region [23]. In China, the National Health Commission regards the number, scale, and proportion of nursing personnel as key indicators of nursing progress [24]. This study draws on official government data, ensuring reliability and minimizing bias. Our analysis reveals significant growth in Jiangxi Province since 2003. Indicators such as the number of registered nurses per 1,000 population, per square kilometers, doctor-to-nurse ratio, and bed-to-nurse ratio ratios have steadily increased. In terms of equity, Jiangxi Province demonstrates absolute equity in the distribution of nursing resources at the population level and reasonable equity at the regional level. The Theil Index results showed that the difference in nursing human resources in Jiangxi Province mainly came from within the region. This suggests that while progress has been made, there is still room for improvement in the regional allocation of nursing human resources. However, Jiangxi Province exhibits a more equitable distribution than the national average. National efforts to improve nursing began in 2005 with the introduction of the outline of the nursing development plan, which is updated every five years. This plan emphasizes optimizing the utilization of nursing graduates to meet healthcare demands. 2008, the Nurse Regulation law further boosted nursing development by legally safeguarding nurses’ rights and mandating that healthcare organizations meet staffing requirements [25]. Rising wages, supported by local policies such as those in Jiangxi Province to protect nurses’ income, have likely contributed to the growing workforce.

Additionally, the New Medical Reform expanded social health insurance coverage to over 97% of the population by 2015 [26], reducing medication costs and increasing medical service demand, indirectly attracting more nurses. With the reform of China’s medical and health system and the deepening of health education, the nursing workforce in China has been significantly developed [5]. In Jiangxi Province, sustained investments in medical security and the implementation of essential public health services at the primary care level have further strengthened nurse recruitment and retention. These policies have improved the quality and efficiency of healthcare while boosting morale among nursing staff [27,28]. These policies focus on enhancing the quality and efficiency of healthcare institutions while optimizing resource utilization [23].

Although some progress in nursing human resource allocation has been made in Jiangxi Province, disparities still exist. In 2022, Jiangxi’s doctor-to-nurse ratio (1:1.27) exceeded the national average (1:1.18) but fell short of the recommended 1:2. The bed-to-nurse ratio in Jiangxi Province (1:0.46) met the recommended standard (1:0.40) but lagged behind the national average (1:0.54). Similarly, Jiangxi’s nurses (3.19 per 1,000 population) were below both the national (3.71) and global averages (3.75) [29]. These imbalances mirror findings in other regions, where differences in medical resources, population density, and economic conditions contribute to unequal nursing resource distribution, aligning with studies from Guangxi Province and other areas [30,31]. Differences may influence such imbalances in medical resources, population density, and regional economic conditions. Despite national efforts to bridge these gaps, resource allocation remains concentrated in densely populated and economically developed areas, leaving underdeveloped regions underserved [14]. As a financially underdeveloped province, Jiangxi Province faces challenges in attracting nursing talent due to lower overall economic and healthcare standards. Factors such as the low social status of the profession, limited recognition, and unclear career advancement paths exacerbate this issue [32]. Legislative measures, such as setting minimum nurse-to-patient ratios and enforcing compliance, could mitigate these disparities [33]. Establishing a nationally standardized salary structure could also help balance resource distribution by addressing dissatisfaction and incentivizing nurses to work in underserved areas [32]. While challenging in a country as vast as China, such initiatives could strengthen the healthcare system and benefit the broader economy.

An analysis of nursing resources across Jiangxi Province’s 11 prefecture-level cities reveals significant disparities. Nanchang City, the provincial capital, has the highest bed-to-nurse ratio in the province and a relatively adequate number of nurses [14]. Smaller cities like Xinyu City have relatively adequate doctor-to-nurse ratios despite their lower economic development. However, the fewest registered nurses reported in Yingtan City. These findings suggest that higher salaries and better job opportunities in wealthier areas only sometimes ensure adequate nursing coverage. In 2017, the urban-county disparity in nursing resources peaked, with urban areas hosting 5.27 registered nurses per 1,000 population compared to just 1.32 in county regions. This shift may have been influenced by Jiangxi Province’s efforts to combat a bird flu outbreak, which likely improved resource allocation and equity [28].

Ongoing provincial initiatives, including annual special funds to revitalize county areas, aim to narrow the urban-county disparity in nursing resources. After 2003, there was notable growth in several key indicators, including the number of registered nurses per thousand inhabitants at both provincial and county levels. The most rapid increase was observed in the number of registered nurses per 1,000 population in Jiangxi Province’s county regions, suggesting that the distribution of nursing human resources in the province is becoming more equitable. This trend promotes the equitable distribution of medical resources, which is beneficial for advancing healthcare accessibility and supporting the development of county medical services. Policies like “Rural region revitalization” have significantly improved healthcare access in county areas [28]. However, this expansion presents new challenges in nursing management, requiring effective strategies to ensure optimal resource utilization. Improving the status of nursing, increasing salaries, and providing more medical resources to underdeveloped regions are essential steps in addressing regional inequities [8,[34], [35], [36]]. Like many other developing countries, China faces a significant nursing shortage [37,38]. Studies from England and the United States indicate inadequate staffing negatively impacts patient care, leading to higher rates of missed nursing interventions, increased burnout, and compromised patient outcomes [30,39]. These issues are similarly pressing in China, which has yet to fully address staffing challenges or establish tools to measure the impact of missed care [[39], [40], [41]]. International programs like the “Happy2Help” (H2H) nurses’ incentive program demonstrate how targeted interventions can enhance caregivers’ job satisfaction [42]. Similarly, applying management tools like operations research techniques and queuing theory to nursing management can improve resource allocation [43,44]. Empowering frontline nursing managers to participate in decision-making and aligning nurse competencies with staffing needs could help reduce attrition and enhance workforce stability [45,46]. Establishing a centralized information platform to manage nurses and healthcare resources is becoming increasingly crucial for effective resource management. As China’s population ages, the allocation of nursing resources in nursing homes and community care settings becomes an urgent priority [47,48]. Global research on nursing home staffing underscores the need to prepare for these demographic challenges. This study employed two prediction models to forecast nursing resource trends in Jiangxi Province. The grey model yielded poor predictive accuracy and was deemed unsuitable for this analysis. The ARIMA model predicted that by 2027, the number of registered nurses per 1,000 population in urban and county areas could reach four or more, exceeding the target (3.80) set in the National Nursing Development Plan (2021–2025). While the urban-county gap is expected to persist, these projections indicate progress toward a more equitable distribution of nursing resources. Establishing a feedback mechanism to capture nurses’ challenges could help reduce turnover and improve job satisfaction, leading to better patient care [49].

## 5 Limitations

The ARIMA model used in this study is limited to univariate time series data, meaning it cannot account for the impact of multiple factors at once. It relies solely on current and past data without incorporating future information, which may reduce the accuracy of long-term predictions. Meanwhile, the Grey model does not apply to this study. For this reason, the study’s forecast extends only to 2027. This study has a single data source, and data from different sources can be collected in the future to verify the accuracy.

## 6 Conclusions

Over the past two decades, nursing resources in Jiangxi Province have grown substantially. The absolute fairness of nurse distribution by population highlights significant progress, although regional disparities persist. Looking ahead, the projected nurse increase will strengthen Jiangxi Province’s capacity to meet the challenges posed by an aging population and unforeseen public health emergencies. This growth will help alleviate medical resource shortages, enhance regional equity in healthcare access, and support the realization of universal health coverage. To fully capitalize on these advancements, China must prioritize improving nursing staff's professional status and compensation. Additionally, leveraging information technology to optimize nurse workforce scheduling and resource management will be essential. By taking these steps, China can ensure a resilient healthcare workforce that protects the well-being of its population and improves health accessibility for all. 10.13039/100014337Furthermore, these efforts will contribute to economic research to optimize the allocation of medical resources, ultimately supporting the country’s healthcare objectives.

## Data availability statement

The datasets generated during and/or analyzed during the current study are available in the two websites: http://www.nhc.gov.cn/mohwsbwstjxxzx/tjzxtjsj/tjsj_list.shtml; https://www.stats.gov.cn/sj/ndsj/.

## CRediT authorship contribution statement

**Yunyu Du:** Conceptualization, Methodology, Writing - original draft, Writing - review & editing. **Zhiqin Xie:** Methodology, Data curation, Writing - review & editing, Supervision. **Zhen Yang:** Conceptualization, Resources, Data curation, Project administration. **Wanyin Xiong:** Methodology, Investigation, Resources. **Li Zhou:** Investigation, Resources, Writing - review & editing. **Min Zhang:** Methodology, Writing - review & editing. **Suhua Zeng:** Conceptualization, Methodology, Supervision. **Min Wang:** Conceptualization, Methodology.

## Funding

None.

## Declaration of competing interest

The authors have declared no conflict of interest.
