# Supplementary material for: Research on equity analysis and forecasting of nursing human resource allocation in Jiangxi Province, China
Source: Int J Nurs Sci. 2024 Dec 19;12(1):19–26. doi: 10.1016/j.ijnss.2024.12.009 (PMC11846548; doi:10.1016/j.ijnss.2024.12.009)
Supplement: Multimedia component 1 [file mmc1.docx]

**中国江西省护理人力资源配置公平性分析与预测研究**

杜韫雨，谢志勤，杨珍，熊万银，周丽，张敏，曾苏华，王敏

【摘要】

**目的** 调查中国江西省护理人力资源的配置现状并评价其公平性，分析未来五年的变化趋势并对需求量进行预测。

**方法** 利用《中国统计年鉴》《中国卫生统计年鉴》和《江西统计年鉴》中2003年至2022年全国以及江西省卫生资源的相关数据，采用洛伦兹曲线、基尼系数与泰尔系数评价护理人力资源分布的公平性，从人口和地理区域两个角度分别进行评价。使用自回归求和移动平均模型和灰色模型预测2023年至2027年江西省护理人力资源的需求量。

**结果** 从2003年至2022年，江西省护理人力资源多个重点指标持续上升。其中，注册护士总人数增加了109 786人；年均增长率为7.80%；每千人口注册护士增加2.21人；每平方公里人口注册护士增加0.66人；医护比从1：0.70提高至1：1.27；床护比从1：0.41提高至1：0.46。江西省注册护士占卫生技术人员的比例、每平方公里人口注册护士人数、医护比均超过全国平均水平。在全省范围内，城市的各项指标均高于县域；赣州市的注册护士数量最多；新余市的医护比最高；南昌市的床护比最高。从人口分布的角度看，江西省注册护士的基尼系数为0.09，达到绝对公平；而在地域分布上，基尼系数为0.34，为相对公平。预测数据显示至2027年，江西省注册护士人数将达到171 000人。

**结论** 2003年至2022年，江西省护理人力资源大幅增长，人口分布具有绝对公平性，地域分布为相对公平。该研究结果为优化江西省未来护理人力资源的分配提供了借鉴价值，为确保患者公平获得医疗保健服务奠定了基础。

【关键词】预测；公平性；护理管理；护理人力资源

通信作者：谢志勤，E-mail：ndyfy09802@ncu.edu.cn
