# Supplementary material for: Research on equity analysis and forecasting of nursing human resource allocation in Jiangxi Province, China
Source: Int J Nurs Sci. 2024 Dec 19;12(1):19–26. doi: 10.1016/j.ijnss.2024.12.009 (PMC11846548; doi:10.1016/j.ijnss.2024.12.009)
Supplement: Multimedia component 2 [file mmc2.docx]

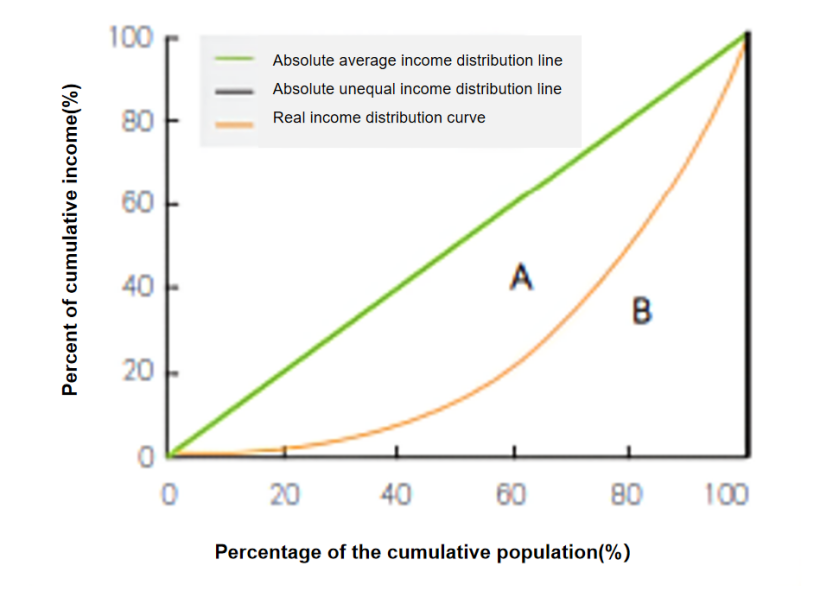


Figure 1. The original figure of the Lorentz curve.


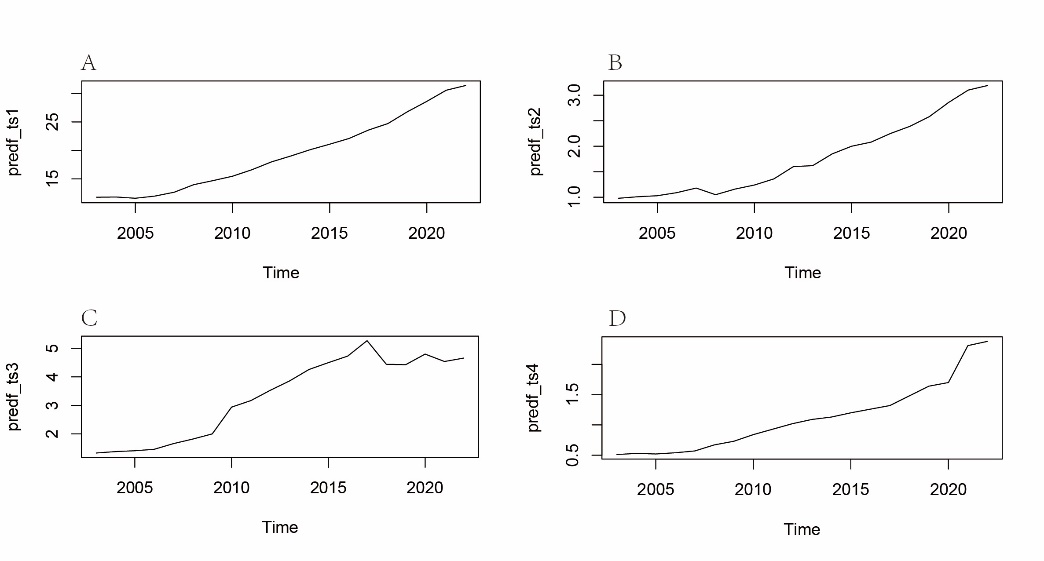


Figure 2. Sequence data line chart (A. Number of registered nurses in Jiangxi Province, B. Number of registered nurses per 1,000 people in Jiangxi, C. Number of registered nurses per 1,000 people in urban area, D. Number of registered nurses per 1,000 people in county seat)


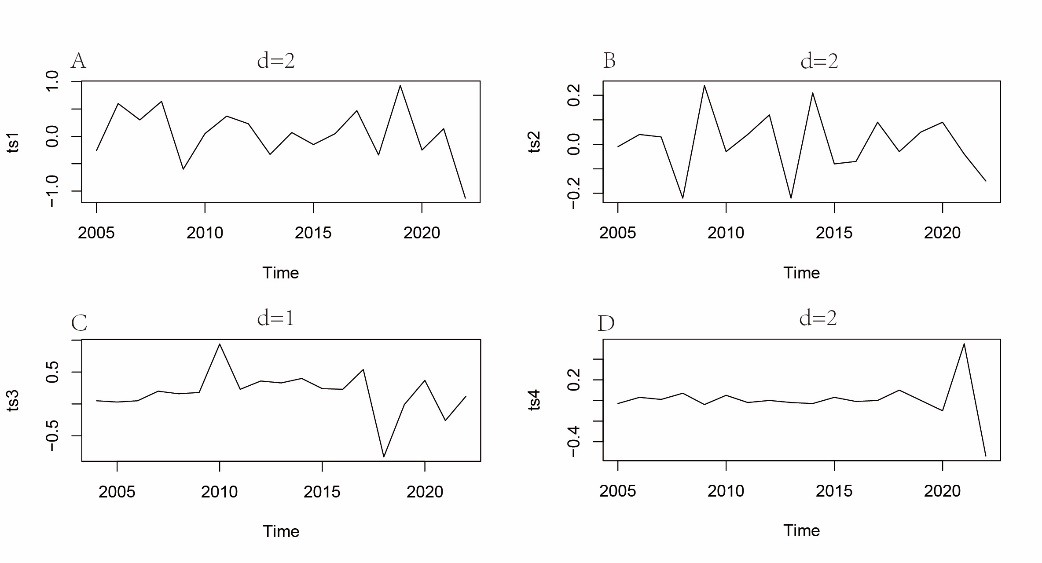


Figure 3. Differential plot of sequence data (A. Number of registered nurses in Jiangxi Province, B. Number of registered nurses per thousand in Jiangxi, C. Number of registered nurses per thousand in urban area, D. Number of registered nurses per thousand in county seat)
