# Supplementary material for: Research on equity analysis and forecasting of nursing human resource allocation in Jiangxi Province, China
Source: Int J Nurs Sci. 2024 Dec 19;12(1):19–26. doi: 10.1016/j.ijnss.2024.12.009 (PMC11846548; doi:10.1016/j.ijnss.2024.12.009)
Supplement: Multimedia component 3 [file mmc3.docx]

**Table 1. Status of Registered Nurse Staffing Nationally and in Jiangxi Province, 2003-2022.**

| Items | Region | 2003 | 2004 | 2005 | 2006 | 2007 | 2008 | 2009 | 2010 | 2011 | 2012 | 2013 | 2014 | 2015 | 2016 | 2017 | 2018 | 2019 | 2020 | 2021 | 2022 |
| --- | --- | --- | --- | --- | --- | --- | --- | --- | --- | --- | --- | --- | --- | --- | --- | --- | --- | --- | --- | --- | --- |
| Health technicians | Nationwide | 4,380,878 | 4,485,983 | 4,564,050 | 4,728,350 | 4,913,186 | 5,174,478 | 5,535,124 | 5,876,158 | 6,202,858 | 6,675,549 | 7,210,578 | 7,589,790 | 8007537 | 8,454,403 | 8,988,230 | 9,529,179 | 10,154,010 | 10,678,019 | 11,244,217 | 11,657,878 |
|  | Jiangxi | 117,755 | 118,196 | 115,986 | 119,761 | 126,598 | 139,764 | 146,990 | 154,733 | 166,069 | 179,797 | 190,234 | 201,327 | 210,946 | 220,979 | 235,773 | 247,204 | 267,917 | 286,089 | 305,670 | 313,,991 |
| Practicing  (assistant) physicians | Nationwide | 1,942,364 | 1,999,457 | 2,042,135 | 2,099,064 | 2,122,925 | 2,201,904 | 2,329,206 | 2,413,259 | 2,466,094 | 2,616,064 | 2,794,754 | 2,892,518 | 3,039,135 | 3,191,005 | 3,390,034 | 3,607,156 | 3,866,916 | 4,085,689 | 4,287,604 | 4,434,728 |
|  | Jiangxi | 49,289 | 46,468 | 46,093 | 51,436 | 51,828 | 55,187 | 56,325 | 59,264 | 62,888 | 67,168 | 70,276 | 74,605 | 76,814 | 79,183 | 83,652 | 87,277 | 96,437 | 104,897 | 111,394 | 113,311 |
| Number of registered nurses | Nationwide | 1,268,959 | 1,308,433 | 1,349,589 | 1,426,339 | 1,558,822 | 1,678,091 | 1,854,818 | 2,048,071 | 2,244,020 | 2,496,599 | 2,783,121 | 3,004,144 | 3,241,469 | 3,507,166 | 3,804,021 | 4,098,630 | 4,445,047 | 4,708,717 | 5,019,422 | 5,224,244 |
|  | Jiangxi | 34,654 | 35,429 | 35,679 | 37,870 | 42,508 | 48,241 | 52,830 | 57,703 | 64,492 | 72,062 | 78,229 | 84,140 | 89,584 | 95,531 | 104,128 | 110,828 | 120,412 | 129,283 | 139,922 | 144,440 |
| Registered nurses as a percentage of health technicians | Nationwide | 0.29 | 0.29 | 0.30 | 0.30 | 0.32 | 0.32 | 0.34 | 0.35 | 0.36 | 0.37 | 0.39 | 0.40 | 0.40 | 0.41 | 0.42 | 0.43 | 0.44 | 0.44 | 0.45 | 0.45 |
|  | Jiangxi | 0.29 | 0.30 | 0.31 | 0.32 | 0.34 | 0.35 | 0.36 | 0.37 | 0.39 | 0.40 | 0.41 | 0.42 | 0.42 | 0.43 | 0.44 | 0.45 | 0.45 | 0.45 | 0.46 | 0.46 |
| Registered nurses per 1,000 population | Nationwide | 1.00 | 1.03 | 1.03 | 1.09 | 1.18 | 1.27 | 1.39 | 1.53 | 1.67 | 1.85 | 2.04 | 2.20 | 2.37 | 2.54 | 2.74 | 2.94 | 3.18 | 3.34 | 3.56 | 3.71 |
|  | Jiangxi | 0.98 | 1.01 | 1.03 | 1.09 | 1.18 | 1.05 | 1.16 | 1.24 | 1.36 | 1.60 | 1.62 | 1.85 | 2.00 | 2.08 | 2.25 | 2.39 | 2.58 | 2.86 | 3.10 | 3.19 |
| Registered nurses per square kilometers | Nationwide | 0.12 | 0.13 | 0.13 | 0.14 | 0.15 | 0.16 | 0.18 | 0.20 | 0.21 | 0.24 | 0.27 | 0.29 | 0.31 | 0.34 | 0.36 | 0.39 | 0.43 | 0.45 | 0.48 | 0.50 |
|  | Jiangxi | 0.21 | 0.21 | 0.21 | 0.23 | 0.25 | 0.29 | 0.32 | 0.35 | 0.39 | 0.43 | 0.47 | 0.50 | 0.54 | 0.57 | 0.62 | 0.66 | 0.72 | 0.77 | 0.84 | 0.87 |
| Doctor-to-nurse ratio | Nationwide | 1:0.65 | 1:0.65 | 1:0.66 | 1: 0.68 | 1: 0.73 | 1: 0.76 | 1:0.80 | 1:0.85 | 1:0.91 | 1:0.95 | 1:1.00 | 1:1.04 | 1:1.07 | 1:1.10 | 1:1.12 | 1:1.14 | 1:1.15 | 1:1.15 | 1:1.17 | 1:1.18 |
|  | Jiangxi | 1:0.70 | 1: 0.76 | 1: 0.77 | 1: 0.74 | 1:0.82 | 1: 0.87 | 1: 0.94 | 1: 0.97 | 1:1.03 | 1:1.07 | 1:1.11 | 1:1.13 | 1:1.17 | 1:1.21 | 1:1.24 | 1:1.27 | 1:1.25 | 1:1.23 | 1:1.26 | 1:1.27 |
| Bed-to-nurse ratio | Nationwide | 1:0.40 | 1:0.40 | 1:0.40 | 1: 0.41 | 1: 0.42 | 1: 0.42 | 1: 0.42 | 1: 0.43 | 1: 0.43 | 1:0.44 | 1:0.45 | 1:0.46 | 1:0.46 | 1: 0.47 | 1: 0.48 | 1: 0.49 | 1:0.50 | 1:0.52 | 1:0.53 | 1:0.54 |
|  | Jiangxi | 1: 0.41 | 1: 0.42 | 1: 0.42 | 1: 0.43 | 1:0.45 | 1:0.46 | 1: 0.43 | 1:0.45 | 1: 0.47 | 1:0.46 | 1:0.45 | 1:0.45 | 1:0.45 | 1:0.46 | 1:0.45 | 1:0.44 | 1:0.45 | 1:0.45 | 1:0.46 | 1:0.46 |

*Note:* Data from Jiangxi Statistical Yearbook 2003-2022 and China Health and Wellness Statistical Yearbook 2003-2022.

**Table 2. Status of Registered Nurse Staffing Across Jiangxi Province.**

| Region | Health technicians | Year-end resident population | Area of jurisdiction (square kilometers) | Number of beds | Practicing  (assistant) physicians | Number of registered nurses | Registered nurses as a percentage of health technicians (%) | Registered nurses per 1,000 population | Registered nurses per square kilometer | Doctor-to-nurse ratio | Bed-to-nurse ratio |
| --- | --- | --- | --- | --- | --- | --- | --- | --- | --- | --- | --- |
| Nanchang  city | 55,191 | 6,538,127 | 7,195 | 45,733 | 19,597 | 26,773 | 0.49 | 4.09 | 3.72 | 1:1.37 | 1:0.59 |
| Jingdezhen  city | 11,725 | 1,621,845 | 5,256 | 11,208 | 4,105 | 5,462 | 0.47 | 3.37 | 1.04 | 1:1.33 | 1: 0.49 |
| Pingxiang  city | 15,007 | 1,808,794 | 3,824 | 14,339 | 5,289 | 7,153 | 0.48 | 3.95 | 1.87 | 1:1.35 | 1:0.50 |
| Jiujiang  city | 31,612 | 4,557,703 | 19,084 | 31,381 | 11,862 | 14,287 | 0.45 | 3.13 | 0.75 | 1:1.20 | 1:0.46 |
| Xinyu  city | 9,568 | 1,202,839 | 3,178 | 9,063 | 3,310 | 4,781 | 0.50 | 3.97 | 1.50 | 1:1.44 | 1:0.53 |
| Yingtan  city | 7,091 | 1,155,837 | 3,556 | 8,063 | 2,600 | 3,125 | 0.44 | 2.70 | 0.88 | 1:1.20 | 1: 0.39 |
| Ganzhou  city | 59,123 | 8,988,068 | 39,379 | 61,999 | 20,911 | 26,714 | 0.45 | 2.97 | 0.68 | 1:1.28 | 1: 0.43 |
| Ji'an  city | 26,948 | 4,422,636 | 25,283 | 31,976 | 10,495 | 11,421 | 0.42 | 2.58 | 0.45 | 1:1.09 | 1: 0.36 |
| Yichun  city | 33,025 | 4,969,735 | 18,669 | 37,521 | 11,835 | 15,024 | 0.45 | 3.02 | 0.80 | 1:1.27 | 1:0.40 |
| Fuzhou  city | 24,669 | 3,578,964 | 18,817 | 22,037 | 9,176 | 11,303 | 0.46 | 3.16 | 0.60 | 1:1.23 | 1: 0.51 |
| Shangrao  city | 40,032 | 6,435,267 | 22,737 | 41,083 | 14,131 | 18,397 | 0.46 | 2.86 | 0.81 | 1:1.30 | 1:0.45 |

*Note:* Data from Jiangxi Statistical Yearbook, 2022.

**Table 3. Comparison of the current status of registered nurses per 1,000 population in cities and county regions in Jiangxi Province, 2003-2022.**

| Items | Geography | 2003 | 2004 | 2005 | 2006 | 2007 | 2008 | 2009 | 2010 | 2011 | 2012 | 2013 | 2014 | 2015 | 2016 | 2017 | 2018 | 2019 | 2020 | 2021 | 2022 |
| --- | --- | --- | --- | --- | --- | --- | --- | --- | --- | --- | --- | --- | --- | --- | --- | --- | --- | --- | --- | --- | --- |
| Health technicians per 1,000 population | City | 3.93 | 4.03 | 4.04 | 4.07 | 4.48 | 4.81 | 5.07 | 6.82 | 7.00 | 7.58 | 8.13 | 8.86 | 9.30 | 9.75 | 10.70 | 8.90 | 8.92 | 9.63 | 9.35 | 9.57 |
|  | County | 2.06 | 2.05 | 1.95 | 1.99 | 1.95 | 2.17 | 2.33 | 2.55 | 2.66 | 2.83 | 2.95 | 3.01 | 3.10 | 3.18 | 3.26 | 3.62 | 3.97 | 4.11 | 5.35 | 5.49 |
| Practicing doctors per 1,000 population | City | 1.60 | 1.65 | 1.66 | 1.70 | 1.78 | 1.86 | 1.93 | 2.58 | 2.51 | 2.64 | 2.79 | 3.05 | 3.10 | 3.29 | 3.60 | 2.97 | 3.02 | 3.32 | 3.29 | 3.35 |
|  | County | 0.89 | 0.90 | 0.87 | 0.88 | 0.82 | 0.87 | 0.95 | 1.02 | 1.04 | 1.10 | 1.14 | 1.17 | 1.20 | 1.19 | 1.20 | 1.33 | 1.50 | 1.58 | 2.01 | 2.04 |
| Registered nurses per 1,000 population | City | 1.33 | 1.38 | 1.41 | 1.46 | 1.66 | 1.82 | 2.00 | 2.94 | 3.17 | 3.53 | 3.86 | 4.26 | 4.50 | 4.73 | 5.27 | 4.44 | 4.43 | 4.80 | 4.54 | 4.66 |
|  | County | 0.51 | 0.53 | 0.52 | 0.54 | 0.57 | 0.67 | 0.73 | 0.84 | 0.93 | 1.02 | 1.09 | 1.13 | 1.20 | 1.26 | 1.32 | 1.48 | 1.64 | 1.70 | 2.31 | 2.38 |

*Note:* Data from Jiangxi Statistical Yearbook, 2003-2022 and China Health Statistics Yearbook, 2003-2022.

**Table 4. Theil index of nursing human resource allocation in Jiangxi Province from 2003 to 2022.**

| Year | Total Theil index | Between-group Theil index | Intra-group Theil index | The Thiel index contribution rate between the groups (%) | The Theil index contribution rate within the group (%) |
| --- | --- | --- | --- | --- | --- |
| 2003 | 0.16 | 0.01 | 0.15 | 4.43 | 95.57 |
| 2004 | 0.15 | 0.01 | 0.14 | 6.61 | 93.39 |
| 2005 | 0.15 | 0.01 | 0.15 | 4.87 | 95.13 |
| 2006 | 0.15 | 0.01 | 0.15 | 4.56 | 95.44 |
| 2007 | 0.15 | 0.01 | 0.14 | 8.01 | 91.99 |
| 2008 | 0.15 | 0.01 | 0.14 | 7.71 | 92.29 |
| 2009 | 0.15 | 0.01 | 0.14 | 8.52 | 91.48 |
| 2010 | 0.15 | 0.01 | 0.14 | 7.97 | 92.03 |
| 2011 | 0.14 | 0.02 | 0.13 | 11.16 | 88.84 |
| 2012 | 0.15 | 0.02 | 0.13 | 12.78 | 87.22 |
| 2013 | 0.15 | 0.02 | 0.13 | 14.59 | 85.41 |
| 2014 | 0.15 | 0.02 | 0.13 | 15.25 | 84.75 |
| 2015 | 0.15 | 0.03 | 0.13 | 18.63 | 81.37 |
| 2016 | 0.15 | 0.03 | 0.12 | 21.21 | 78.79 |
| 2017 | 0.16 | 0.04 | 0.13 | 23.43 | 76.57 |
| 2018 | 0.17 | 0.04 | 0.13 | 24.02 | 75.98 |
| 2019 | 0.17 | 0.04 | 0.14 | 22.56 | 77.44 |
| 2020 | 0.18 | 0.04 | 0.14 | 21.93 | 78.07 |
| 2021 | 0.18 | 0.04 | 0.14 | 23.12 | 76.88 |
| 2022 | 0.18 | 0.04 | 0.14 | 23.84 | 76.16 |

Table 5 ARIMA model and Grey (1,1) model predictions of nursing human resources in Jiangxi Province

| Year | Registered number of registered nurses | | | Registered nurses per 1,000 population | | | Registered nurses per 1,000 population in urban areas | | | Registered nurses per 1,000 population in county | | |
| --- | --- | --- | --- | --- | --- | --- | --- | --- | --- | --- | --- | --- |
|  | Original value | ARIMA  (0,2,0) | Grey (1,1) model | Original value | ARIMA  (0,2,1) | Grey (1,1) model | Original value | ARIMA  (0,1,0) | Grey (1,1) model | Original value | ARIMA  (2,2,0) | Grey (1,1) model |
| 2003 | 3.47 | - | 3.47 | 0.98 | - | 0.98 | 1.33 | - | 1.33 | 0.51 | - | 0.51 |
| 2004 | 3.54 | - | 2.81 | 1.01 | - | 0.73 | 1.38 | - | 1.51 | 0.53 | - | 0.32 |
| 2005 | 3.57 | 3.63 | 3.31 | 1.03 | 1.05 | 0.84 | 1.41 | 1.56 | 1.70 | 0.52 | 0.57 | 0.41 |
| 2006 | 3.79 | 3.62 | 3.82 | 1.09 | 1.07 | 0.95 | 1.46 | 1.59 | 1.91 | 0.54 | 0.57 | 0.49 |
| 2007 | 4.25 | 4.03 | 4.34 | 1.18 | 1.15 | 1.06 | 1.66 | 1.64 | 2.10 | 0.57 | 0.59 | 0.58 |
| 2008 | 4.82 | 4.73 | 4.89 | 1.05 | 1.25 | 1.17 | 1.82 | 1.84 | 2.30 | 0.67 | 0.62 | 0.67 |
| 2009 | 5.28 | 5.41 | 5.45 | 1.16 | 1.09 | 1.29 | 2.00 | 2.00 | 2.51 | 0.73 | 0.73 | 0.76 |
| 2010 | 5.77 | 5.76 | 6.02 | 1.24 | 1.22 | 1.41 | 2.94 | 2.18 | 2.72 | 0.84 | 0.85 | 0.85 |
| 2011 | 6.45 | 6.28 | 6.61 | 1.36 | 1.31 | 1.53 | 3.17 | 3.12 | 2.93 | 0.93 | 0.96 | 0.94 |
| 2012 | 7.21 | 7.15 | 7.22 | 1.60 | 1.45 | 1.65 | 3.53 | 3.35 | 3.15 | 1.02 | 1.07 | 1.03 |
| 2013 | 7.82 | 7.99 | 7.84 | 1.62 | 1.72 | 1.78 | 3.86 | 3.71 | 3.37 | 1.09 | 1.17 | 1.13 |
| 2014 | 8.41 | 8.45 | 8.49 | 1.85 | 1.74 | 1.91 | 4.26 | 4.04 | 3.59 | 1.13 | 1.24 | 1.22 |
| 2015 | 8.96 | 9.02 | 9.15 | 2.00 | 1.99 | 2.04 | 4.50 | 4.44 | 3.82 | 1.20 | 1.27 | 1.32 |
| 2016 | 9.55 | 9.53 | 9.83 | 2.08 | 2.15 | 2.18 | 4.73 | 4.68 | 4.08 | 1.26 | 1.3 | 1.42 |
| 2017 | 10.41 | 10.16 | 10.53 | 2.25 | 2.23 | 2.31 | 5.27 | 4.91 | 4.28 | 1.32 | 1.37 | 1.52 |
| 2018 | 11.08 | 11.29 | 11.25 | 2.39 | 2.41 | 2.46 | 4.44 | 5.45 | 4.52 | 1.48 | 1.44 | 1.63 |
| 2019 | 12.04 | 11.77 | 11.99 | 2.58 | 2.56 | 2.60 | 4.43 | 4.62 | 4.76 | 1.64 | 1.57 | 1.73 |
| 2020 | 12.93 | 13.02 | 12.76 | 2.86 | 2.76 | 2.75 | 4.80 | 4.61 | 5.00 | 1.70 | 1.79 | 1.84 |
| 2021 | 13.99 | 13.84 | 13.54 | 3.10 | 3.05 | 2.90 | 4.54 | 4.98 | 5.25 | 2.31 | 1.94 | 1.94 |
| 2022 | 14.44 | 15.07 | 14.35 | 3.19 | 3.31 | 3.06 | 4.66 | 4.72 | 5.50 | 2.38 | 2.35 | 2.05 |
| 2023 |  | 14.91 | 15.18 |  | 3.40 | 3.21 |  | 4.84 | 5.76 |  | 2.84 | 2.16 |
| 2024 |  | 15.40 | 16.04 |  | 3.62 | 3.38 |  | 5.01 | 6.02 |  | 3.19 | 2.28 |
| 2025 |  | 15.92 | 16.92 |  | 3.85 | 3.54 |  | 5.19 | 6.28 |  | 3.49 | 2.39 |
| 2026 |  | 16.45 | 17.82 |  | 4.09 | 3.71 |  | 5.36 | 6.55 |  | 3.97 | 2.51 |
| 2027 |  | 17.01 | 18.75 |  | 4.33 | 3.89 |  | 5.54 | 6.82 |  | 4.32 | 2.63 |

*Note:* ARIMA = Autoregressive Integrated Moving Average.

Table 6 Grey (1,1) model and test results.

| Forecast project | Parameter values | Fits equation | Test statistic *C* | *P* | Model precision rank |
| --- | --- | --- | --- | --- | --- |
| Number of registered nurses | a = -0.028  u = 164969.01 | X^(1)^(*k*)=(3.47+5891750.357) *e*^0.028（^*^k^*^-1）^-5891750.357 | 0.004 | 0.55 | 4 Level |
| Number of registered nurses per 1,000 population | a = -0.022  u = 4.568 | X^(1)^(*k*)=(0.98+207.636) *e*^0.022（^*^k^*^-1）^-207.636 | 0.034 | 0.35 | 4 Level |
| Number of registered nurses per 1,000 population in urban areas | a = -0.015  u = 12.218 | X^(1)^(*k*)=(1.33+814.533) *e*^0.015（^*^k^*^-1）^-814.533 | 0.137 | 0.40 | 4 Level |
| Number of registered nurses per 1,000 people in the County regions | a = -0.016  u = 5.195 | X^(1)^(*k*)=(0.51+324.688) *e*^0.016（^*^k^*^-1）^-324.688 | 0.073 | 0.55 | 4 Level |

Table 7 Comparison of absolute relative error (%) of prediction results between Grey (1,1) and ARIMA models

| Year | Number of registered nurses | | Number of registered nurses per 1,000 population | | Number of registered nurses per 1,000 population in urban | | Number of registered nurses per 1,000 population in county regions | |
| --- | --- | --- | --- | --- | --- | --- | --- | --- |
|  | ARIMA  (0,2,0) | Grey (1,1) | ARIMA  (0,2,1) | Grey (1,1) | ARIMA  (0,1,0) | Grey (1,1) | ARIMA  (2,2,0) | Grey (1,1) |
| 2003 |  | 0 | - | 0 | - | 0 | - | 0 |
| 2004 |  | 20.772 | - | 27.373 | - | 9.023 | - | 39.054 |
| 2005 | 1.681 | 7.368 | 1.942 | 18.879 | 10.638 | 20.477 | 9.615 | 21.756 |
| 2006 | 4.485 | 0.804 | 1.835 | 12.827 | 8.904 | 29.859 | 5.556 | 8.880 |
| 2007 | 5.176 | 2.208 | 2.542 | 10.409 | 1.205 | 26.279 | 3.509 | 1.502 |
| 2008 | 1.867 | 1.306 | 19.048 | 11.479 | 1.099 | 26.354 | 7.463 | 0.531 |
| 2009 | 2.462 | 3.071 | 6.034 | 10.917 | 0.000 | 25.310 | 0.000 | 3.522 |
| 2010 | 0.173 | 4.318 | 1.613 | 13.334 | 25.850 | 7.621 | 1.190 | 0.759 |
| 2011 | 2.636 | 2.497 | 3.676 | 12.259 | 1.577 | 7.603 | 3.226 | 0.912 |
| 2012 | 0.832 | 0.165 | 9.375 | 3.177 | 5.099 | 10.898 | 4.902 | 1.179 |
| 2013 | 2.174 | 0.263 | 6.173 | 9.736 | 3.886 | 12.824 | 7.339 | 3.399 |
| 2014 | 0.476 | 0.867 | 5.946 | 3.106 | 5.164 | 15.772 | 9.735 | 8.281 |
| 2015 | 0.670 | 2.127 | 0.500 | 2.005 | 1.333 | 15.229 | 5.833 | 10.135 |
| 2016 | 0.209 | 2.900 | 3.365 | 4.603 | 1.057 | 14.487 | 3.175 | 12.794 |
| 2017 | 2.402 | 1.135 | 0.889 | 2.863 | 6.831 | 18.815 | 3.788 | 15.330 |
| 2018 | 1.895 | 1.527 | 0.837 | 2.770 | 22.748 | 1.705 | 2.703 | 9.804 |
| 2019 | 2.243 | 0.393 | 0.775 | 0.821 | 4.289 | 7.375 | 4.268 | 5.456 |
| 2020 | 0.696 | 1.324 | 3.497 | 3.867 | 3.958 | 4.196 | 5.294 | 7.970 |
| 2021 | 1.072 | 3.214 | 1.613 | 6.420 | 9.692 | 15.637 | 16.017 | 15.880 |
| 2022 | 4.363 | 0.648 | 3.762 | 4.203 | 1.288 | 18.075 | 1.261 | 13.759 |

*Note:* ARIMA: Autoregressive Integrated Moving Average.
